# Fruit Odor as A Ripeness Signal for Seed-Dispersing Primates? A Case Study on Four Neotropical Plant Species

**DOI:** 10.1007/s10886-016-0687-x

**Published:** 2016-04-02

**Authors:** Omer Nevo, Eckhard W. Heymann, Stefan Schulz, Manfred Ayasse

**Affiliations:** Behavioral Ecology and Sociobiology Unit, German Primate Center, Kellnerweg 4, 37077 Göttingen, Germany; Department of Sociobiology/Anthropology, Johann-Friedrich-Blumenbach Institute for Zoology and Anthropology, Georg-August University of Göttingen, Kellnerweg 6, 37077 Göttingen, Germany; Institute of Evolutionary Ecology and Conservation Genomics, University of Ulm, Helmholtzstr. 10-1, Containerstadt, 89081 Ulm, Germany; Institute of Organic Chemistry, Technische Universität Braunschweig, Hagenring 30, 38106 Braunschweig, Germany

**Keywords:** Coevolution, Dispersal syndromes, Fruit secondary metabolites, Fruit odor, Mutualism, Olfaction

## Abstract

Fleshy fruits contain a myriad of secondary metabolites that may fulfill various non-mutually exclusive ecological functions. Among them are defense against pathogens and herbivores, manipulation of frugivores’ gut retention time, or controlling the germination process. In addition, it has been suggested that aroma compounds may be used as fruit-selection cues by frugivores, and that plants may be under selection to provide a reliable signal for ripeness to seed-dispersal vectors through ripe fruit aroma. A previous project demonstrated that fruit odor of two Neotropical primate-dispersed plant species can be used by primates to identify ripe fruits. Here, we provide data supporting the hypothesis that olfactory conspicuousness of ripeness in these two species may be an evolved signal rather than a cue exploited by primates. We analyzed the odors of ripe and unripe fruits of the two species along with odors of two sympatric species whose main dispersal vector is passerine birds. We show that only primate-dispersed species significantly change their odor profiles upon ripening. Thus, odor of bird-dispersed species is not informative regarding their ripeness level and is likely to fulfill other functions. We discuss these data in light of the multiple hypotheses for the presence of fruit secondary metabolites, and we offer a roadmap for future studies to establish the hypothesis that fruit odor is an evolved signal for ripeness.

**Electronic supplementary material:**

The online version of this article (doi:10.1007/s10886-016-0687-x) contains supplementary material, which is available to authorized users.

## Introduction

Plants synthesize over 100,000 different secondary metabolites (PSMs) that fulfill a myriad of functions (Knudsen et al. 2006). In vegetative parts, the main function of PSMs is direct or indirect defense against pathogens and herbivores. Flowers and fruits also utilize PSMs for defense. However, in many species, they also interact with animal vectors of pollination and seed dispersal, and hence their PSM profiles are subjected to multidirectional, sometimes conflicting, selection pressures. While many questions remain unresolved, the biochemistry, ecology, and evolution of floral fragrance, i.e., their volatile PSM profiles, have received a great deal of attention over the past decades. In contrast, inquiries into the nature of fruit PSMs, especially volatiles, have lagged behind.

### Why Do Fruits Contain Secondary Metabolites?

Several hypotheses have been put forward to explain the presence of fruit PSMs. A commonly held null hypothesis is that their presence derives directly from synthesis of defensive compounds in vegetative parts or unripe fruits and thus requires little further explanation (Eriksson and Ehrlén 1998). However, PSM profiles of ripe fruits are not strictly correlated with those of leaves or unripe fruits (Whitehead and Bowers 2013). This indicates that fruit PSMs may go beyond pleiotropy.

Cipollini and Levey (1997) suggested several non-mutually exclusive adaptive functions of fruit PSMs. As in vegetative organs, PSMs can play a defensive role. They can have general toxicity and thus also deter mutualists (the *defense trade-off* hypothesis). For example, iridoid glycosides in a hybrid bush honeysuckle defend fruits against pathogens and insects but may deter birds – a major seed disperser (Whitehead and Bowers 2013). Alternatively, PSMs can be toxic to antagonists without deterring legitimate dispersers (*directed toxicity*). For instance, capsaicinoids in chilli peppers (*Capsicum* spp.) deter mammalian seed predators without decreasing the fruits’ palatability to seed-dispersing birds (Tewksbury and Nabhan 2001). Nonetheless, an *a-priori* assumption that fruit PSMs play a defensive role is unwarranted. For example, terpenoids often are considered to be defensive compounds. However, downregulation of limonene (a monoterpene) synthesis in *Citrus* fruits renders the fruits *less* susceptible to microbial pathogens and less attractive to invertebrate antagonists (Rodríguez et al. 2011).

Somewhat ironically, the *attraction*/*repulsion* hypothesis postulates that defensive PSMs may be directed against legitimate vertebrate dispersal vectors in order to prevent excessive feeding by a single seed disperser and subsequent deposition of the seeds in a clump. A variation of the attraction/repulsion hypothesis is the *protein assimilation* hypothesis, according to which PSMs may inhibit protein assimilation by frugivores and thus promote further movement and deposition of the seeds away from the mother tree. These hypotheses have so far received little support.

Ripe fruit PSMs also can *manipulate the frugivore*’*s gut retention time*, thus maximizing dispersal distance while avoiding excessive damage during digestion. While few studies have demonstrated that some fruit PSMs alter frugivores’ gut-retention time, none has unequivocally established a connection to an increased fitness benefit (e.g., Wahaj et al. 1998). In addition, PSMs may function in *inhibiting seed germination*. This ensures that the seeds do not germinate prematurely, and the removal of the pulp by frugivores triggers germination after the dispersal event (Cipollini and Levey 1997). Several studies have reported inhibiting effects of either individual compounds, fruit pulp, or pulp extracts. However, the effect is not universal (a compound which inhibits germination in one species does not necessarily have the same effect in others), and it is not always clear whether inhibition has a positive effect on individual plants’ fitness (e.g., Wahaj et al. 1998).

### PSMs as Seed-Disperser Attractants

Fleshy fruits are selected to be attractive, and PSMs also have been suggested to act as frugivore attractants. The *attraction*/*association* hypothesis postulates that PSMs may provide cues regarding fruit ripeness or quality. They can do so through visual cues (pigments such as carotenoids and anthocyanins) or via the chemical senses through aroma compounds. Candidates for such compounds are those whose biosynthesis is associated directly with desired macronutrients (Cipollini and Levey 1997). For example, nitrogen-containing compounds derive from amino acid metabolism (Knudsen et al. 2006) and can therefore provide an honest signal for protein content. In this case, it is difficult to distinguish between cues, which provide information but are not necessarily selected to fulfill this function, and signals, which are selected to convey information.

Plant secondary metabolites also may act as signals directed at frugivores even without direct biochemical association with the reward. For instance, lipid content in a community of Mediterranean fleshy fruits is positively correlated with fruit color, which in turn promotes consumption by birds (Schaefer et al. 2014). A similar process has been observed in pollination systems (Schiestl 2015). The logic here is that generalist mutualists can learn to prefer plants that provide a signal and “punish” cheaters. Thus, through repeating interactions over evolutionary time-scales, reliable signals may be selected. Aroma compounds, which reliably signal a fruit’s ripeness and promote ingestion and further foraging on the same plant, may even be selected to be present in the pulp rather than the husk. They may be favored in fruits whose thick, leathery or non-permeable husk constrains efficient odor emission.

### Volatile PSMs – Fruit Odor as a Signal for Vertebrate Dispersal Vectors

The hypothesis that fruit odor, its volatile PSMs profile, can be a signal to seed dispersers is not new. In practice, however, it rarely has been addressed in either empirical studies or theoretical considerations. For example, in their thorough book on plant-animal communication, Schaefer and Ruxton (2011) discuss the potential roles of ethylene and ethanol as odor cues, not signals. The strongest evidence that plants employ volatile PSMs to signal ripeness to frugivores comes from a comparison of different fig species (*Ficus* spp.). The odor of ripe bat-dispersed figs is attractive to them, and thus mediates the communication between the plants and their primary seed dispersers (Hodgkison et al. 2013). Bat-dispersed figs also change odor profiles upon ripening, which allows bats to easily detect and identify the ripe fruits; at the same time, bird-dispersed species do not show a pronounced shift in odor at ripeness, thus indicating that the shift of odor in bat-dispersed figs is not a trait that characterizes all figs but only those which rely on olfactory-guided bats for seed dispersal (Borges et al. 2008). Finally, bat-dispersed fig species tend to emit higher amounts of odor than do bird-dispersed figs (Lomáscolo et al. 2010). Overall, this mirrors the patterns known in pollination ecology: bat-pollinated flowers tend to be odiferous, while bird-pollinated flowers are usually scentless. Otherwise, studies addressing this question are practically absent, and most data available on ripe fruit odor bouquets come from cultivated species and is therefore less relevant.

In a recent project on the role of primate olfaction in fruit selection, we showed that spider monkeys (*Ateles geoffroyi*), Neotropical frugivorous primates that are important seed dispersers, can readily discriminate between odors of ripe and unripe (husk or pulp) fruits of two primate-dispersed Neotropical species, *Couma macrocarpa* (Apocynaceae) and *Leonia cymosa* (Violaceae) (Nevo et al. 2015). This is consistent with the observation that in food acquisition, primates employ their sense of smell mainly for quality assessment of individual fruits (Nevo and Heymann 2015). Primates are important seed dispersers in the tropics, and in the Neotropics they constitute a significant part of a more-or-less discrete mammalian dispersal syndrome. Thus, there are many fruiting species that rely on the dispersal services of primates and other arboreal mammals, and they may be a suitable model system to test the hypothesis that the odors used by primates are not only cues but evolved signals.

Here, we present some data supporting this hypothesis. We compared patterns of odor emission between *C. macrocarpa* and *L. cymosa* and two sympatric bird-dispersed species, under the assumption that passerine-dispersed fruits tend to signal through visual rather than olfactory signals. We sampled fruit odors of ripe and unripe, intact and open (husk and pulp odor) fruits of the two primate-dispersed species and of bird-dispersed *Maieta guianensis* (Melastomataceae) and *Psychotria cincta* (Rubiaceae). Compound identity and concentration were analyzed by using gas-chromatography coupled with mass-spectrometry. In each species, we examined whether odor profiles of ripe and unripe fruits are significantly different from one another, either at the intact (husk odor) or open (pulp odor) conditions. The logic is that due to the various functions of fruit PSMs, fruits of all species, ripe and unripe, are expected to emit at least trace amounts of odor. Crucially, most non-signaling functions can be achieved by a similar odor bouquet whereas signaling to seed dispersers requires a substantial change in the odor profile to reliably signal ripeness. Thus, we predicted that if odor has evolved as a signal for vertebrates, a significant shift in the odor profile upon ripening is expected *only in species that rely on olfactory-guided primates*.

## Methods and Materials

Fruits from different trees were enclosed in unused oven bags (Toppits, Germany). After 2.5 hr, their headspace was collected for 10 min onto a self-produced absorbent Chromatoprobe trap (1.5 mg Tenax-TA, 1.5 mg Carbotrap. Both Supelco, Sigma-Aldrich, Germany). Samples were kept frozen in −20°c and analyzed on a Hewlett Packard 6890 Series gas chromatographic–mass selective detector (GC–MS; Agilent Quadrupol 5972) equipped with a DB-5 ms capillary column (30 m long, 250 μm diam, film thickness: 0.25 μm, J&W). Sample size was *N* = 9 to *N* = 15 fruits per species/condition. Compounds were tentatively identified by comparing their mass spectra and retention indices with published data. Identity of the majority of dominant compounds was confirmed by running synthetic compounds in identical conditions. Data were analyzed for each species separately. Principal component analyses were used to reduce the number of variables in the dataset and eliminate collinearity between them. Then, PCs which cumulatively explained at least 90 % of the original variance were used in discriminant function analyses and MANOVA tests, which examined whether the overall odor bouquet of ripe and unripe fruits differ either in the intact (husk odor) or open (pulp odor) conditions, and thus is potentially informative to frugivores. More details on the study system, sampling and analyses are available as online supplementary materials.

## Results

**Fig. 1:**
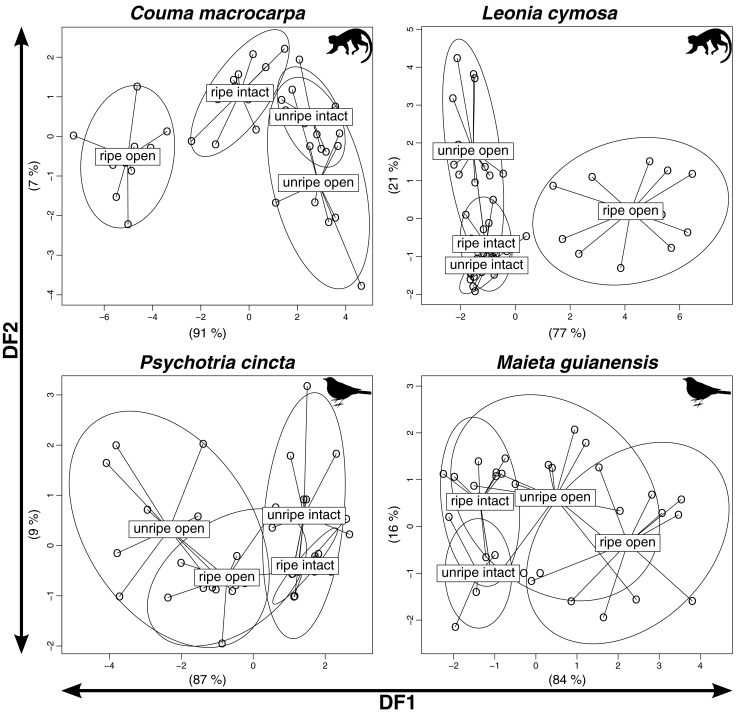
Discriminant function analyses (DFAs) to estimate the discrimination potential between odor profiles of ripe and unripe fruits of each species. *DFAs* were conducted on principal components that accounted for at least 90 % of the original variance (*Couma macrocarpa*: 8 PCs, *Leonia cymosa*: 7, *Psychotria cincta*: 5, *Maieta guianensis*: 4). Numbers adjacent to axes are the proportion of between-group variance explained by the respective discriminant function. Clearly distinct odor profile of ripe fruits (intact, open or both) is present only in primate-dispersed species (for test statistics, see results)

Discriminant function analyses and subsequent *MANOVA* tests revealed clear differences between odor bouquets of ripe and unripe primate-dispersed fruits, but not in the two bird-dispersed species. In the bird-dispersed *P. cincta*, the discriminant function analysis (*DFA*) produced overlapping clusters, and the odor of ripe fruits was indistinguishable from that of unripe fruit in both intact and open conditions (Fig. 1). The first linear discriminant function (*DF1*) significantly separated odor samples from the four conditions in the species (ripe/unripe, intact/open) (*Wilks*’ *lambda* = 0.18, *chi-square* = 59.09 (15), *P* < 0.001) while *DF2* did not (*Wilks*’ *lambda* = 0.69, *chi-square* = 12.71 (8), *P* = 0.12). Consequently, *MANOVA* tests could not discriminate between the odor profiles of ripe and unripe fruits in either intact (*N* = 21, *F*(4, 16) = 1.69, *adjusted P* = 1) or open (*N* = 19, *F*(4, 14) = 2.16, *adjusted P* = 1) conditions. Similarly, odor profiles of ripe and unripe *M. guianensis* fruits showed strong overlap in either intact or open conditions. Discrimination was stronger, and both *DFs* were significant (*DF1*: Wilks’ lambda =0.2, *chi-square* = 58.53 (12), *P* < 0.001; *DF2*: *Wilks*’ *lambda* = 0.68, *chi-square* = 14.05 (6), *P* = 0.03). However, discrimination between odor profiles of ripe and unripe fruits was still low (intact: *N* = 20, *F*(4, 15) = 4.9, *adjusted P* = 0.08. Open: *N* = 21, *F*(4, 16) = 3.31, *adjusted P* = 0.3). Thus, in either intact or open conditions, ripe and unripe bird-dispersed species emitted odors that are similar and did not provide reliable information regarding their ripeness level.

In contrast, *DFAs* in primate-dispersed species generated clearly separate clusters of ripe and unripe fruits. In *C. macrocarpa*, the *DFA* as a whole was significant (*DF1*: *Wilks*’ *lambda* = 0.39, *chi-square* = 110.58 (24), *P* < 0.001; *DF2*: *Wilks*’ *lambda* = 0.46, *chi-square* = 26.41 (14), *P* = 0.02) (Fig. 1), and the odor profiles of ripe and unripe fruits were significantly different in both intact (*N* = 21, *F*(8, 12) = 8.33, *adjusted P* < 0.01) and open conditions (*N* = 20, *F*(8, 11) = 22.6, *adjusted P* < 0.001). In *L. cymosa* (*DF1*: *Wilks*’ *lambda* = 0.052, *chi-square* = 125.33 (21), *P* < 0.001; *DF2*: *Wilks*’ *lambda* = 0.35, *chi-square* = 44.95 (12), *P* < 0.001) the odor profiles of intact ripe and unripe fruits were indistinguishable (*N* = 25, *F*(7, 17) = 2.01, *adjusted P* = 0.9), but the odor of open ripe fruits formed a cluster significantly different from the odor of unripe open fruits (*N* = 24, *F*(7, 16) = 12.63, *adjusted P* < 0.001). Thus, in both species, ripe fruits were characterized by odors that are unique to the ripe phase and are indicative of their ripeness level.

## Discussion

Ripe fruits of the two primate-dispersed species emit odors that are significantly different from odors of conspecific unripe fruits (husk, pulp, or both), which renders their aromas unique to the ripe phase and reliably convey information to primates, their main dispersal vector (Nevo et al. 2015). In contrast, fruits of the two bird-dispersed species emit odors that are indistinguishable from the odors of unripe fruits. Passerines have elaborated visual capacities, and fruits they disperse tend to provide conspicuous visual cues (Lomáscolo and Schaefer 2010). Thus, it is possible that *M. guianensis* and *P. cincta* have been under selection to focus their signaling efforts on the visual cues. This pattern is similar to bird-pollinated flowers, which often emit only trace amounts of odors and provide conspicuous visual signals. A possible function of the volatile compounds identified is defense, since in contrast to primate-dispersed species, in order to be available to small understory passerines these and similar fruits are soft and only lightly protected by a protective husk. Thus, they are more susceptible to pathogens and herbivores than *C. macrocarpa* and *L. cymosa*, which are protected by a thick husk. Other alternative explanations may also explain the presence of these volatiles in the two bird-dispersed species.

Each of the primate-dispersed species is phylogenetically closer to one of the bird-dispersed species than they are to one another (see online supplementary materials). Taken together, these results indicate that the olfactory conspicuousness – the substantial shift in odor profile upon ripeness - of *C. macrocarpa* and *L. cymosa* may not be an inevitable characteristic of fleshy-fruit maturation, which can be used by primates as a cue for fruit selection, but possibly an evolved signal whose function is to convey information to seed-dispersing primates. At the same time, these conclusions are based on only four species, and it is impossible to extrapolate from them to the entire system. While they indicate that signaling ripeness to seed-dispersing mammals may be an evolved function of fruit odor, this hypothesis remains to be established.

### How to Continue? A Roadmap for Inferring whether Fruit Odor Is an Evolved Signal for Ripeness

Inferring adaptation is never a straightforward task, especially in multidimensional traits such as fruit odor, and when various, non-mutually exclusive, alternative explanations are applicable. We suggest three approaches that together can establish the hypothesis that signaling to vertebrates is one of the functions PSMs fulfill in some taxa: the ecological, comparative and integrative approaches.

- *Ecological Approach* Before asking whether fruit odor is an evolved signal directed at vertebrates, it must be established that it is at the very least used as a cue, i.e., that seed-dispersal vectors rely on it in the process of food selection. This can be done both in observational studies that quantify sniffing behavior of frugivores in the wild, or in controlled experiments that annihilate other cues and ask whether the vertebrate can identify ripe fruits in the absence of cues from other sensory modalities. A second prediction that should be confirmed is that these olfactory cues are not redundant in the more natural scenario when cues from other sensory modalities are available. Here, an effective experimental design could present vertebrates with choice tests between items that provide visual cues, olfactory cues, or a combination of both. The expectation is that the presence of odor provides non-redundant information regarding fruit maturity, and hence the combination of olfactory and visual cues should be preferred over visual cues alone. Finally, the hypothesis that some components of fruit odor have evolved, or are maintained by selection by vertebrate frugivores also requires that these vertebrates would show consistent preference for fruits with a more pronounced signal.
- *Comparative Approach* A second phase in establishing that that fruit odor is an evolved signal for ripeness is to demonstrate its absence in plant lineages that rely on dispersal services by animals that are less likely to use olfaction in fruit selection. Given the alternative adaptive and non-adaptive explanations for the presence of fruit PSMs, all fruits are likely to emit some VOCs (volatile organic compounds). Thus, the prediction in a comparative study would not be that fruits whose primary seed dispersers are not olfactory-guided do not have an odor at all, but rather that their odors are substantially less informative than those of fruits that depend on olfactory-guided vertebrates. Hence, the main prediction should be that all other things being equal, the former emit odor bouquets that are not significantly different between ripe and unripe fruits, while the latter show a pronounced shift in the VOC profile upon ripening. While the data presented above provide some support that this is the case in four Neotropical species, confirmation of this hypothesis would require a larger dataset that would allow quantitative phylogenetic-controlled analyses.
- *Integrative Approach* Finally, another approach that would help in establishing whether the odors vertebrates use in fruit selection are signals rather than cues is an examination, and exclusion, of alternative explanations. Once it is established that fruit odor mediates the communication between seed dispersers and plants (i.e., that it is a cue), exclusion of alternative hypotheses would increase the probability that a major selection pressure for the presence of ripe fruits VOCs is selection by frugivores. For example, biochemical pleiotropy can be excluded if odor profiles of ripe fruits are significantly different, quantitatively or qualitatively, from VOC profiles of other plant organs and unripe fruits. Adaptive functions can be tested using various designs. Antibiotic properties of odor components can be estimated using classical antibiotic tests or through genetic manipulation of odor profiles and comparison of the susceptibility of wild-type and genetically-modified fruits to various antagonists. Other functions can be tested using both correlational studies of natural populations or controlled experiments.
  This approach, however, suffers from several shortcomings. First, fruit volatiles may fulfill many different functions, some not yet conceived, and eliminating all might prove impossible. Second, in many cases, it is impossible to fully exclude a function. For example, a study can show that certain VOCs in ripe fruits are not effective defenders against pathogens that are known to attack the fruit. However, it still could be that these substances are effective against another pathogen that has not even been identified as an antagonist of the plant species due to its effective defense mechanisms. In this case, a highly effective defensive compound may actually be considered to have no defensive properties. Third, PSMs may act in concert with other PSMs or require specific conditions that are difficult to replicate in controlled experiments. Finally, PSMs could fulfill various non-mutually exclusive functions, or their contemporary main function could be a secondary adaptation. For example, floral odorants that convey information to pollinating invertebrates are believed to have originally evolved as defensive barriers. Thus, filtering out the relative role of each function on the presence of a certain compound or compound group is neither sufficient nor straightforward.

In summary, fruit PSMs have been suggested to fulfill various, non-mutually-exclusive functions. An attractive, yet under-investigated, hypothesis is that their role is to signal ripeness to seed-dispersing vertebrates. The data presented here provide some support for this idea. However, to establish this hypothesis many factors need to be controlled. We suggest three approaches which, together, may allow one to disentangle this complex question, and we hope that future studies will take this path to provide more established answers to the question whether fleshy fruits whose main dispersal vectors are olfactory-dependent vertebrates have evolved to communicate with them via olfactory signals.

## Electronic Supplementary Material

ESM 1 (DOCX 791 kb)
