# Supplementary material for: Fruit Odor as A Ripeness Signal for Seed-Dispersing Primates? A Case Study on Four Neotropical Plant Species
Source: J Chem Ecol. 2016 Apr 2;42:323–8. doi: 10.1007/s10886-016-0687-x (PMC4869761; doi:10.1007/s10886-016-0687-x)
Supplement: Supplementary file 1 — (DOCX 791 kb) [file 10886_2016_687_MOESM1_ESM.docx]

**SUPPLEMENTARY MATERIALS**

Fruit odor as a ripeness signal for seed-dispersing primates? A case study on four Neotropical plant species

Journal of Chemical Ecology

Omer Nevo* ^1,2,3^, Eckhard W. Heymann^1^, Stefan Schulz^4^ & Manfred Ayasse^3^

*1. Behavioral Ecology and Sociobiology Unit, German Primate Center, Kellnerweg 4, 37077 Göttingen, Germany*

*2. Department of Sociobiology / Anthropology, Johann-Friedrich-Blumenbach Institute for Zoology and Anthropology, Georg-August University of Göttingen, Kellnerweg 6, 37077 Göttingen, Germany*

*3. Institute of Evolutionary Ecology and Conservation Genomics, University of Ulm, Helmholtzstr. 10-1, Containerstadt, 89081 Ulm, Germany*

*4. Institute of Organic Chemistry, Technische Universität Braunschweig, Hagenring 30, 38106 Braunschweig, Germany*

**Contents:**

- Methods and materials
- Additional results:
  - Tab. S1-S4: compounds found in the four model species in all conditions.
  - Fig S1-S4: representative gas chromatograms of samples from all species and conditions.
- Bibliography for Supp Mat.

**Methods and materials**

**Model System.** Odor samples of fruits were collected at the Estación Biológica Quebrada Blanco, Loreto, north-eastern Peru (4°21’S 73°09’W), between February and June 2013 (for more details on the site, see Heymann 1995). Due to the difficulties of obtaining a sufficient amount of samples in the tropics, we restricted our analyses to four model species. Model species were selected during field work because it is impossible to predict which species would provide fruits in sufficient amounts in a given year. We selected species that were abundant and dyssynchronous (i.e., provided both ripe and unripe fruits at the same time). This was important for both technical and theoretical reasons: first, to allow sampling in both modes under the same conditions, and second, because in fully synchronous species, signals for ripeness are probably less important since there is no need to distinguish ripe from unripe fruits. We selected species that allowed us to control for phylogeny and that are either primate or bird dispersed. *Couma macrocarpa* (Apocynaceae) is a canopy tree whose fruits are yellow, globular, and large (> 4 cm diam) and dispersed by many primate species (Culot et al. 2009; Peres 1994). *Leonia cymosa* (Violaceae) is an understory tree producing yellow globular fruits (ca. 3 cm diam) dispersed exclusively by primates (Pfrommer 2009; Reinehr 2010). Fruits of both species are protected with a thick husk preventing access from small birds. *Psychotria cincta* (Rubiaceae) provides small (ca. 0.5 cm diam) soft orange fruits. Fruits of this genus are dispersed by small birds (Gorchov et al. 1995; Snow 1981). *Maieta guianensis* (Melastomataceae) provides small (ca. 1 cm diam) dark and soft berries like most other fleshy-fruit producing Melastomataceae. Apart from a single genus (*Bellucia*), all fleshy fruits from this family are dispersed by birds (Renner 1989; Stiles and Rosselli 1993). Both species were observed to be consumed by small understory passerines (O.N., pers. obs.) and are usually inaccessible to primates because they grow on small bushes or treelets. Long-term behavioral and ecological field work on tamarin monkeys (*Saguinus mystax*, *Saguinus nigrifrons*), which are smaller than most other Neotropical primates, confirmed that *P. cincta* and *M. guianensis* are never consumed by primates even when they have physical access to the fruits (Culot 2009; Knogge and Heymann 2003; Smith 1997). Finally, all four model species are from families that include species dispersed by the other dispersal vector (i.e., primates in the case of the two bird-dispersed species and vice versa) (Ballard and Sytsma 2000; Culot et al. 2010; Julliot and Sabatier 1993; Wheelwright et al. 1984) as well as species producing non-endozoochoric dry fruits (Ballard et al. 2014; Bolmgren and Eriksson 2005; Bremer and Eriksson 1992; Clausing et al. 2000). Thus, while we cannot provide any information regarding ancestral states, fruit traits in these families showed, over evolutionary time, malleability that allowed many lineages to adapt in response to selection pressures exerted by different dispersal vectors. Each primate-dispersed species is phylogenetically closer to one bird-dispersed species than they are to one another (Davies et al. 2004).

**Collection of Fruit Odor Samples.** Fresh ripe and unripe fruits were collected from different trees/shrubs, cleaned with a wet cloth, and sampled; each fruit individually, within up to 4 h of removal. *P. cincta* infructescences often contain small clusters of 2-3 ripe or unripe fruits, which means that if it signals ripeness via the olfactory channel, it may be a combined signal of more than one fruit. We, thus, always sampled two fruits from the same infructescense at a time from this species. Within species, sampling in the four conditions (ripe/unripe, intact/open) was unordered, although for natural reasons, ripe fruits (intact/open) tended to be sampled later in the season. For sampling of the pulp odor (open fruits), fruits were cut through with a clean knife. Fruits were enclosed in chambers of 30 cm unused inert oven bags (Toppits, Germany) for 2.5 h. Then, headspace odors were collected for 10 min in a constant airflow of 330 ml/min onto a self-made adsorbent trap containing 3 mg 1:1 mixture of Tenax-TA and Carbotrap (both Supelco, Sigma-Aldrich, Germany) (Dötterl et al. 2005). Adsorbent traps were installed on a cleaned (ethanol EMSURE 99.9% and pentane EMSURE 99%, Merck, Germany) Teflon tube that penetrated the chamber and was the only opening in the system. Adsorbent traps were packed individually in clean 1.5 ml vials sealed with Teflon caps (Supelco, Sigma-Aldrich, Germany) and held frozen in -20°C until analysis apart from a 4-d transportation period in which they were packed isolated with freezing packs (Techni-ice, Australia) frozen to -30°C. Sample size was 9-15 fruits per species, and condition (ripe or unripe, intact or open), totaling at *N* = 40 – 49 samples per species in all conditions. To identify possible contaminations, we took at least one control sample (same conditions, chamber without a fruit) on every sampling day.

**Chemical Analyses.** Samples were analyzed on a Hewlett Packard HP 6890 Series gas chromatographic–mass selective detector (GC–MS; Agilent Quadrupol 5972) equipped with a DB-5ms capillary column (30 m long, 250 µm diam, film thickness: 0.25 µm, J&W) which had been fitted with the ChromatoProbe kit (Dötterl and Jürgens 2005). An adsorbent trap was loaded into the probe, which was inserted into the modified GC injector at an injector temperature of 300 °C. Volatile traps were injected splitless at an oven temperature of 40°C. After 1 min, the split valve was opened, and the oven temperature was increased by 6°C / min until reaching 120°C and then by 10°C / min until reaching 310°C, a temperature that was held for 6 extra min. Tentative identification of most compounds was carried out using the Adams (2007) mass spectral data bases on MSD Chemstation (build 75) and confirmed by comparison of their retention indices with those from the published libraries (Adams 2007; NIST 11). Retention indices were calculated using an external alkane standard (C9 – C31) which was run at the same conditions several times. We confirmed the identity of the majority of dominant compounds (see Tab. S1-S4) by running known synthetic compounds at the same conditions. Compounds were quantified using Amdis 2.71. Absolute amounts were estimated using an external standard of 0.01 mg *p*-Cymene which we ran 10 times under identical conditions.

**Statistical Analysis.** Before further analyses, in each species, we excluded compounds which were present in less than eight samples of that species and compounds considered as contamination. We considered a compound to be a contaminant when it was present in similar concentrations in the control samples. Few compounds (e.g., limonene) were contaminants but also appeared in much bigger concentrations in *C. macrocarpa*, which implied that it was a genuine peak in this species. We thus excluded it from the species in which the concentration was on par with that of control samples, but kept it for *C. macrocarpa*.

To compare the odor profiles of ripe and unripe fruits in either intact or open conditions, we first, for each species separately, applied a principal component analysis (*PCA*) to reduce the number of variables and avoid collinearity between them. Since *C. macrocarpa* showed a high number of minor compounds, in order to avoid inflating the number of variables in the statistical tests we used only compounds whose absolute amounts exceeded 100 ng in at least one sample. We then used the PCs that accounted for at least 90% of the original variance (*C. macrocarpa*: 8 PCs, *L. cymosa*: 7, *P. cincta*: 5, *M. guianensis*: 4) as new variables in discriminant function analyses (*DFA*) applied for each species separately. We used DFAs to graphically examine, for each species, whether odor profiles of ripe and unripe fruits in both conditions (intact and open) form separate clusters. To answer the biologically relevant question whether odor is indicative of the ripeness level of the fruits and quantify the level of discrimination between the clusters of ripe and unripe fruits (intact or open) identified in the DFAs, we applied a *post-hoc* analysis comprised of a series of 8 pair-wise *MANOVA* tests. *MANOVA* tests are mathematically identical to *DFA* (Quinn and Keough 2002) and thus allow using the same data for this analysis in which, for each species, we tested whether the odor profiles of ripe and unripe intact fruits are significantly different from one another, and similarly whether profiles of open ripe and unripe fruits are different. *P*-values then were subjected to the Bonferroni correction for multiple testing. All analyses were conducted on R 3.0.3 (R Core Team 2014) with packages MASS (Venables and Ripley 2002) and ade4 (Dray and Dufour 2007) and on SPSS v. 22.

**Tab. S1: - Compounds identified from *Couma macrocarpa.*** Mean (±SD) estimated absolute amounts (mg) of compounds in all *Couma macrocarpa* samples in mg after 2.5 h sampling and retention indices (RI). Absolute amounts were calculated based on an external standard. Values are rounded and thus very small amounts are sometimes presented as 0. Compounds presented are those present in at least 8 samples in a species, including the minor compounds (< 100 ng in at least one sample) that were considered in the odor richness analysis but not odor production, PCA and DFA (see Methods). ID method: methods used for identification of the respective compound. RI: retention index matching published values. MS: strong match with published mass spectra. REF: compound identification verified by running a synthetic compound or, in cases of enantiomers and close derivatives, a mixture of known compounds. In unknown compounds, ID method provides dominant MS ions. Compound classes: terpenoids (TRP), aromatic compounds (AR), aldehydes (ALD), alcohols (ALC), esters (EST), acids (AC).

| **Code** | **ID** | **Class** | **RI** | **ID method** | **Ripe intact** | **Unripe intact** | **Ripe open** | **Unripe open** |
| --- | --- | --- | --- | --- | --- | --- | --- | --- |
| C1 | Cumene | AR | 9.23 | RI, MS, REF | 0.004 ±(0.007) | 0.003 ±(0.007) | 0.01 ±(0.027) | 0.009 ±(0.029) |
| C2 | α-Thujene | TRP | 9.25 | RI, MS | 0.001 ±(0.002) | 0.003 ±(0.007) | 0.001 ±(0.004) | 0.025 ±(0.061) |
| C3 | α-Pinene | TRP | 9.32 | RI, MS | 0.051 ±(0.068) | 0.028 ±(0.044) | 0.094 ±(0.19) | 0.119 ±(0.276) |
| C4 | Benzaldehyde | AR, ALD | 9.63 | RI, MS, REF | 0.01 ±(0.011) | 0.001 ±(0.003) | 0.189 ±(0.441) | 0.011 ±(0.02) |
| C5 | Sabinene | TRP | 9.73 | RI, MS | 0.001 ±(0.003) | 0.027 ±(0.046) | 0 ±(0.001) | 0.03 ±(0.04) |
| C6 | Myrcene | TRP | 9.89 | RI, MS, REF | 0.023 ±(0.021) | 0.063 ±(0.1) | 0.047 ±(0.042) | 0.068 ±(0.084) |
| C7 | α-Terpinene | TRP | 10.17 | RI, MS | 0 ±(0) | 0.008 ±(0.014) | 0 ±(0.001) | 0.022 ±(0.041) |
| C8 | *p*-Cymene | AR, TRP | 10.25 | RI, MS, REF | 0.004 ±(0.005) | 0.029 ±(0.038) | 0.011 ±(0.008) | 0.094 ±(0.161) |
| C9 | Limonene | TRP | 10.30 | RI, MS, REF | 0.026 ±(0.019) | 0.274 ±(0.418) | 0.057 ±(0.051) | 0.258 ±(0.339) |
| C10 | (*E*)-β−Ocimene | TRP | 10.47 | RI, MS, REF | 0.067 ±(0.105) | 0.014 ±(0.015) | 0.116 ±(0.208) | 0.025 ±(0.031) |
| C11 | γ−Terpinene | TRP | 10.58 | RI, MS, REF | 0.008 ±(0.025) | 0.018 ±(0.029) | 0.001 ±(0.002) | 0.028 ±(0.052) |
| C12 | Acetophenone | AR | 10.67 | RI, MS, REF | 0.002 ±(0.003) | 0.001 ±(0.002) | 0.003 ±(0.004) | 0.001 ±(0.003) |
| C13 | *cis*-Linalool oxide (furanoid) | TRP, ALC | 10.71 | RI, MS | 0.002 ±(0.004) | 0 ±(0) | 0.016 ±(0.042) | 0.012 ±(0.014) |
| C14 | Unknown |  | 10.74 | Dominant ions: 43, 85, 69, 58 | 0.003 ±(0.005) | 0.017 ±(0.039) | 0.006 ±(0.006) | 0.011 ±(0.026) |
| C15 | Unknown monoterpene | TRP | 10.86 | Dominant ions: 121, 91, 79, 105, 77, 41, 93, 136, 119 | 0 ±(0) | 0.003 ±(0.006) | 0.001 ±(0.003) | 0.01 ±(0.018) |
| C16 | *trans*-Linalool oxide (furanoid) | TRP, ALC | 10.87 | RI, MS | 0.001 ±(0.004) | 0 ±(0) | 0.016 ±(0.042) | 0.009 ±(0.011) |
| C17 | *p*-Cymenene | AR, TRP | 10.91 | RI, MS, REF | 0 ±(0) | 0.005 ±(0.007) | 0 ±(0) | 0.015 ±(0.028) |
| C18 | Unknown |  | 10.93 | Dominant ions: 89, 41 | 0.001 ±(0.002) | 0.003 ±(0.006) | 0.003 ±(0.007) | 0.015 ±(0.02) |
| C19 | Methyl benzoate | AR, EST | 10.96 | RI, MS | 0.001 ±(0.003) | 0 ±(0) | 0.009 ±(0.012) | 0 ±(0) |
| C20 | Unknown |  | 10.98 | Dominant ions: 55, 41, 69, 84, 96 | 0 ±(0) | 0 ±(0) | 0.022 ±(0.023) | 0 ±(0) |
| C21 | Linalool | TRP, ALC | 11.01 | RI, MS, REF | 0.014 ±(0.027) | 0.002 ±(0.007) | 0.065 ±(0.158) | 0.077 ±(0.111) |
| C22 | (*Z*)-6-Nonenal | ALD | 11.03 | RI, MS | 0 ±(0) | 0 ±(0) | 0.033 ±(0.039) | 0 ±(0) |
| C23 | Nonanal | ALD | 11.06 | RI, MS, REF | 0.02 ±(0.014) | 0.015 ±(0.012) | 0.106 ±(0.065) | 0.013 ±(0.013) |
| C24 | (*E*)-4,8-Dimethyl-1,3,7-nonatriene | TRP | 11.14 | RI, MS | 0.01 ±(0.014) | 0.024 ±(0.042) | 0.054 ±(0.107) | 0.152 ±(0.185) |
| C25 | *allo*-Ocimene | TRP | 11.29 | RI, MS, REF | 0.011 ±(0.014) | 0.003 ±(0.006) | 0.019 ±(0.025) | 0.01 ±(0.013) |
| C26 | (2*E*,6*Z*)-2,6-Nonadienal | ALD | 11.53 | RI, MS | 0.002 ±(0.004) | 0 ±(0) | 0.077 ±(0.073) | 0.001 ±(0.002) |
| C27 | *Trans*-2-nonenal | ALD | 11.61 | RI, MS, REF | 0.008 ±(0.014) | 0 ±(0) | 0.213 ±(0.169) | 0.002 ±(0.007) |
| C28 | Methyl salicylate | AR | 11.93 | RI, MS, REF | 0.034 ±(0.057) | 0 ±(0.001) | 0.269 ±(0.506) | 0.011 ±(0.013) |
| C29 | Methyl 6-nonenoate | EST | 12.21 | RI, MS | 0.01 ±(0.025) | 0 ±(0) | 0.02 ±(0.01) | 0 ±(0) |
| C30 | Methyl nonanoate | EST | 12.25 | RI, MS | 0.003 ±(0.009) | 0 ±(0) | 0.01 ±(0.006) | 0 ±(0) |
| C31 | Unknown |  | 12.29 | Dominant ions: 123, 43, 180 | 0 ±(0.001) | 0.003 ±(0.008) | 0 ±(0.001) | 0.002 ±(0.006) |
| C32 | Unknown |  | 12.31 | Dominant ions: 93, 43, 121, 41, 40, 121, 108, 136, 55, 79, 66, 151, 166 | 0 ±(0) | 0 ±(0) | 0.006 ±(0.011) | 0.001 ±(0.001) |
| C33 | Ethyl salicylate | AR | 12.71 | RI, MS, REF | 0.015 ±(0.029) | 0 ±(0) | 0.079 ±(0.089) | 0 ±(0) |
| C34 | Neryl formate | TRP | 12.83 | RI, MS | 0.002 ±(0.004) | 0 ±(0) | 0.013 ±(0.011) | 0 ±(0) |
| C35 | α-Cubebene | TRP | 13.50 | RI, MS | 0.123 ±(0.129) | 0.122 ±(0.152) | 0.116 ±(0.112) | 0.111 ±(0.126) |
| C36 | α-Ylangene | TRP | 13.73 | RI, MS | 0.004 ±(0.007) | 0.006 ±(0.011) | 0.007 ±(0.01) | 0.012 ±(0.017) |
| C37 | α-Copaene | TRP | 13.80 | RI, MS, REF | 0.68 ±(0.459) | 0.74 ±(0.764) | 0.776 ±(0.757) | 1.21 ±(1.19) |
| C38 | β-Bourbonene | TRP | 13.87 | RI, MS | 0.003 ±(0.003) | 0.002 ±(0.003) | 0.003 ±(0.003) | 0.001 ±(0.001) |
| C39 | β-Cubebene | TRP | 13.90 | RI, MS | 0.013 ±(0.013) | 0.01 ±(0.014) | 0.01 ±(0.009) | 0.022 ±(0.026) |
| C40 | β-Elemene | TRP | 13.91 | RI, MS | 0.012 ±(0.019) | 0.014 ±(0.025) | 0.015 ±(0.023) | 0.001 ±(0.004) |
| C41 | Unknown |  | 14.00 | Dominant ions: 189, 57, 43, 133, 91, 107, 71, 148, 204 | 0.002 ±(0.006) | 0.004 ±(0.01) | 0.005 ±(0.008) | 0.004 ±(0.007) |
| C42 | (*Z*)-Caryophyllene | TRP | 14.07 | RI, MS, REF | 0.007 ±(0.007) | 0.001 ±(0.002) | 0.025 ±(0.018) | 0.003 ±(0.01) |
| C43 | α-Funebrene | TRP | 14.14 | RI, MS, REF | 0.001 ±(0.003) | 0.004 ±(0.007) | 0.001 ±(0.004) | 0.001 ±(0.002) |
| C44 | (*E*)-Caryophyllene | TRP | 14.24 | RI, MS, REF | 0.235 ±(0.189) | 0.073 ±(0.091) | 0.69 ±(0.452) | 0.057 ±(0.054) |
| C45 | β-Copaene | TRP | 14.34 | RI, MS | 0.005 ±(0.003) | 0.007 ±(0.008) | 0.007 ±(0.008) | 0.015 ±(0.02) |
| C46 | α-Bergamotene | TRP | 14.35 | RI, MS | 0.001 ±(0.003) | 0.009 ±(0.014) | 0 ±(0.001) | 0.002 ±(0.007) |
| C47 | Unknown sesquiterpene | TRP | 14.54 | RI | 0.003 ±(0.004) | 0.008 ±(0.011) | 0.011 ±(0.015) | 0.008 ±(0.009) |
| C48 | Unknown sesquiterpene | TRP | 14.59 | MS | 0.006 ±(0.015) | 0.01 ±(0.025) | 0.014 ±(0.026) | 0.008 ±(0.015) |
| C49 | α-Humulene | TRP | 14.61 | RI, MS, REF | 0.021 ±(0.017) | 0.012 ±(0.016) | 0.069 ±(0.039) | 0.007 ±(0.008) |
| C50 | γ-Muurolene | TRP | 14.78 | RI, MS | 0.003 ±(0.003) | 0.003 ±(0.004) | 0.006 ±(0.009) | 0.011 ±(0.024) |
| C51 | Unknown |  | 14.86 | Dominant ions: 41, 105, 91, 69, 162, 119, 159 | 0.001 ±(0.003) | 0.009 ±(0.018) | 0.001 ±(0.002) | 0.006 ±(0.01) |
| C52 | β-Selinene | TRP | 14.94 | RI, MS | 0.077 ±(0.078) | 0.101 ±(0.134) | 0.073 ±(0.054) | 0.037 ±(0.035) |
| C53 | α-Selinene | TRP | 15.00 | RI, MS, REF | 0.033 ±(0.033) | 0.04 ±(0.052) | 0.032 ±(0.027) | 0.02 ±(0.023) |
| C54 | (*E*,*E*)-α-Farnesene | TRP | 15.05 | RI, MS, REF | 0.032 ±(0.07) | 0.194 ±(0.363) | 0.035 ±(0.073) | 0.141 ±(0.258) |
| C55 | δ-Amorphene | TRP | 15.22 | RI, MS | 0.012 ±(0.012) | 0.012 ±(0.016) | 0.018 ±(0.013) | 0.021 ±(0.024) |
| C56 | *cis*-Calamenene | TRP | 15.26 | RI, MS | 0.007 ±(0.01) | 0.003 ±(0.004) | 0.007 ±(0.009) | 0.013 ±(0.028) |
| C57 | *trans*-Cadina-1,4-diene | TRP | 15.38 | RI, MS | 0.003 ±(0.005) | 0.004 ±(0.006) | 0.004 ±(0.005) | 0.004 ±(0.006) |
| C58 | α-Calacorene | TRP, AR | 15.48 | RI, MS | 0.001 ±(0.002) | 0.001 ±(0.001) | 0.002 ±(0.003) | 0.005 ±(0.011) |
| C59 | Unknown sesquiterpene | TRP | 15.89 | RI | 0 ±(0) | 0 ±(0) | 0.021 ±(0.02) | 0 ±(0) |
|  | TOTAL |  |  |  | 1.45 ±(0.86) | 1.77 ±(1.82) | 3.08 ±(1.29) | 2.41 ±(2.21) |


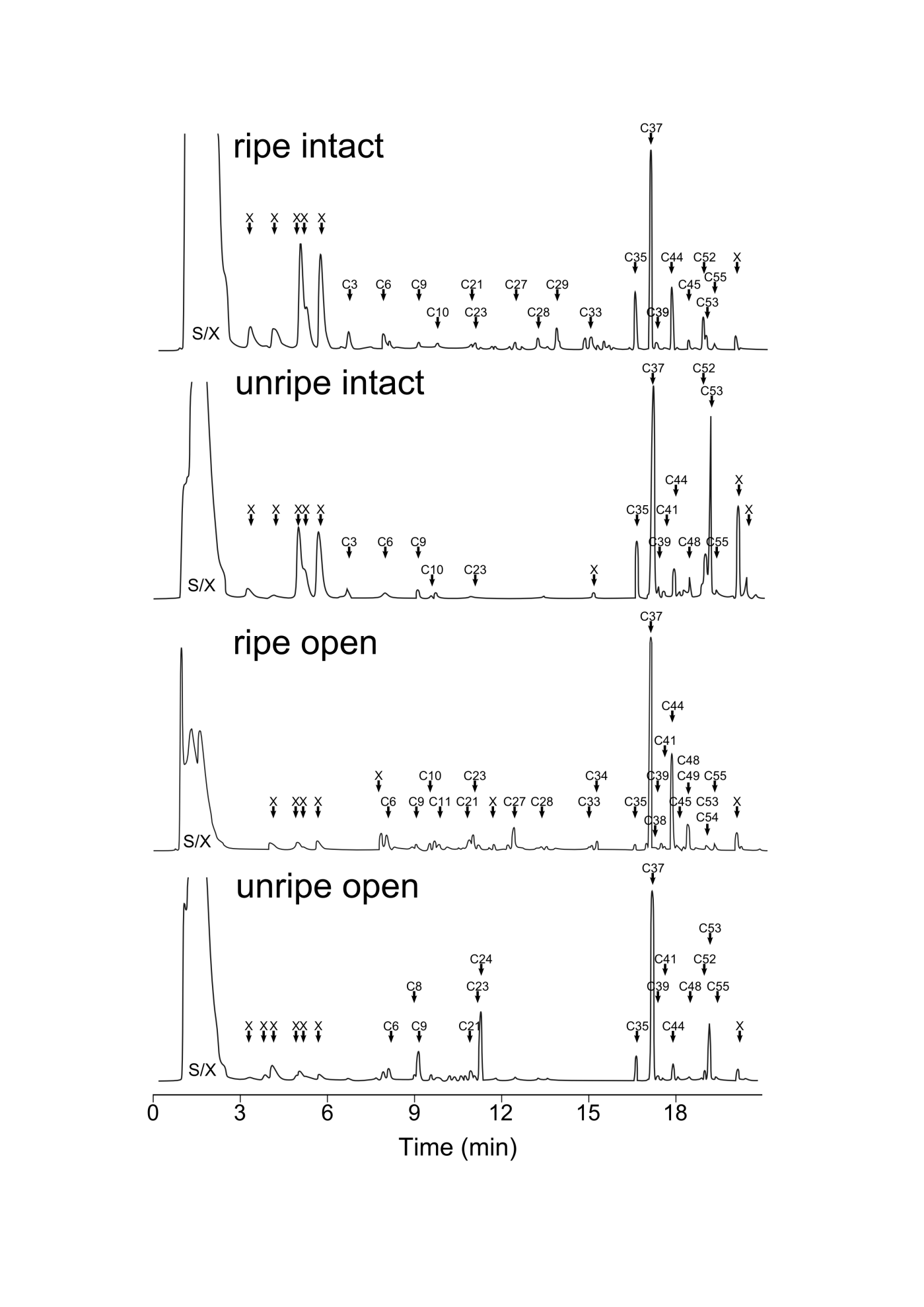


**Fig. S1 – Representative gas chromatograms of odor profiles of *Couma macrocarpa*.** Compound names are shown in Tab. S1. X marks contaminants. SX marks a solvent & unknown contaminant peaks present in all samples, including control. Presented samples were chosen according to their proximity to the group means (e.g., ripe intact). However, they may markedly deviate from the group mean with regards to some individual compounds and are thus for illustration only.

**Tab. S2: Compound identified from *Leonia cymosa.*** Mean (±SD) estimated absolute amounts (mg) of compounds in all *Leonia cymosa* samples in mg after 2.5 h sampling and retention indices (RI). Absolute amounts were calculated based on an external standard. Values are rounded and thus very small amounts are sometimes presented as 0. Compounds presented are those present in at least 8 samples in a species (see Methods). ID method: methods used for identification of the respective compound. RI: retention index matching published values. MS: strong match with published mass spectra. REF: compound identification verified by running a synthetic compound or, in cases of enantiomers and close derivatives, a mixture of known compounds. In unknown compounds, ID method provides dominant MS ions.

| **Code** | **ID** | **Class** | **RI** | **ID method** | **Ripe intact** | **Unripe intact** | **Ripe open** | **Unripe open** |
| --- | --- | --- | --- | --- | --- | --- | --- | --- |
| L1 | Cumene | AR | 9.23 | RI, MS, REF | 0.001 ±(0.002) | 0.013 ±(0.023) | 0.011 ±(0.034) | 0.018 ±(0.054) |
| L2 | Benzaldehyde | AR, ALD | 9.63 | RI, MS, REF | 0.014 ±(0.019) | 0.004 ±(0.009) | 0.062 ±(0.153) | 0.05 ±(0.153) |
| L3 | Myrcene | TRP | 9.89 | RI, MS, REF | 0 ±(0) | 0 ±(0) | 0.025 ±(0.037) | 0.008 ±(0.028) |
| L4 | Unknown monoterpene | TRP | 10.06 | RI, MS | 0 ±(0) | 0 ±(0) | 1.083 ±(1.302) | 0 ±(0) |
| L5 | *p*-Cymene | AR, TRP | 10.25 | RI, MS, REF | 0.003 ±(0.004) | 0.001 ±(0.001) | 0.02 ±(0.043) | 0.009 ±(0.032) |
| L6 | (*Z*)-β−Ocimene | TRP | 10.36 | RI, MS, REF | 0 ±(0) | 0 ±(0) | 0.043 ±(0.059) | 0.003 ±(0.011) |
| L7 | (*E*)-β−Ocimene | TRP | 10.49 | RI, MS, REF | 0.006 ±(0.008) | 0 ±(0) | 5.999 ±(7.201) | 0.009 ±(0.01) |
| L8 | Acetophenone | AR | 10.67 | RI, MS, REF | 0.006 ±(0.006) | 0.008 ±(0.01) | 0.009 ±(0.009) | 0.003 ±(0.004) |
| L9 | 2,6-Dimethyl-1,3,5,7-octatetraene | TRP | 10.81 | RI, MS | 0.001 ±(0.002) | 0.002 ±(0.004) | 0.091 ±(0.091) | 0.001 ±(0.003) |
| L10 | Unknown |  | 10.87 | Dominant ions: 91, 105, 77, 119 | 0 ±(0) | 0 ±(0) | 0.031 ±(0.038) | 0 ±(0) |
| L11 | *p*-Cymenene | AR | 10.92 | RI, MS, REF | 0 ±(0) | 0 ±(0) | 0.199 ±(0.207) | 0.001 ±(0.002) |
| L12 | *p*-1,3,8-Menthatriene | TRP | 11.18 | RI, MS | 0 ±(0) | 0 ±(0) | 0.171 ±(0.234) | 0 ±(0) |
| L13 | *allo*-Ocimene | TRP | 11.29 | RI, MS, REF | 0.001 ±(0.003) | 0 ±(0) | 0.37 ±(0.62) | 0.012 ±(0.028) |
| L14 | *neo-allo*-Ocimene | TRP | 11.41 | RI, MS, REF | 0 ±(0) | 0 ±(0) | 0.133 ±(0.187) | 0.001 ±(0.002) |
| L15 | (*Z*)-3-hexenyl 2-methylbutyrate | EST | 12.32 | RI, MS | 0 ±(0) | 0 ±(0) | 0.002 ±(0.004) | 0.016 ±(0.025) |
| L16 | α-Cubebene | TRP | 13.50 | RI, MS | 0.01 ±(0.025) | 0.002 ±(0.007) | 0.009 ±(0.016) | 0.031 ±(0.033) |
| L17 | α-Ylangene | TRP | 13.73 | RI, MS | 0.008 ±(0.024) | 0.001 ±(0.002) | 0.012 ±(0.017) | 0.036 ±(0.081) |
| L18 | α-Copaene | TRP | 13.79 | RI, MS, REF | 0.043 ±(0.106) | 0.007 ±(0.009) | 0.06 ±(0.085) | 0.237 ±(0.535) |
| L19 | Unknown sesquiterpene | TRP | 13.90 | RI, MS | 0.002 ±(0.008) | 0 ±(0.002) | 0.005 ±(0.009) | 0.013 ±(0.02) |
| L20 | α-Funebrene | TRP | 14.13 | RI, MS, REF | 0 ±(0.001) | 0.004 ±(0.006) | 0.003 ±(0.005) | 0.005 ±(0.01) |
| L21 | β-Copaene <beta-> | TRP | 14.34 | RI, MS | 0.004 ±(0.009) | 0 ±(0) | 0.008 ±(0.012) | 0.02 ±(0.052) |
| L22 | γ-Muurolene | TRP | 14.78 | RI, MS | 0.001 ±(0.004) | 0.001 ±(0.003) | 0.003 ±(0.004) | 0.007 ±(0.02) |
| L23 | α-Muurolene | TRP | 15.01 | RI, MS | 0.004 ±(0.014) | 0 ±(0.002) | 0.017 ±(0.018) | 0.027 ±(0.059) |
| L24 | δ-Amorphene | TRP | 15.21 | RI, MS | 0.003 ±(0.007) | 0.001 ±(0.002) | 0.013 ±(0.014) | 0.017 ±(0.022) |
| L25 | *cis*-Calamenene | TRP | 15.26 | RI, MS | 0.001 ±(0.002) | 0 ±(0.001) | 0.002 ±(0.002) | 0.006 ±(0.011) |
| L26 | *trans*-Cadina-1,4-diene | TRP | 15.37 | RI, MS, REF | 0.001 ±(0.002) | 0 ±(0) | 0.002 ±(0.003) | 0.003 ±(0.004) |
| L27 | α-Calacorene | TRP, AR | 15.47 | RI, MS | 0 ±(0.001) | 0 ±(0) | 0.001 ±(0.001) | 0.002 ±(0.002) |
|  | TOTAL |  |  |  | 0.11 ±(0.21) | 0.04 ±(0.04) | 8.38 ±(9.07) | 0.53 ±(0.86) |


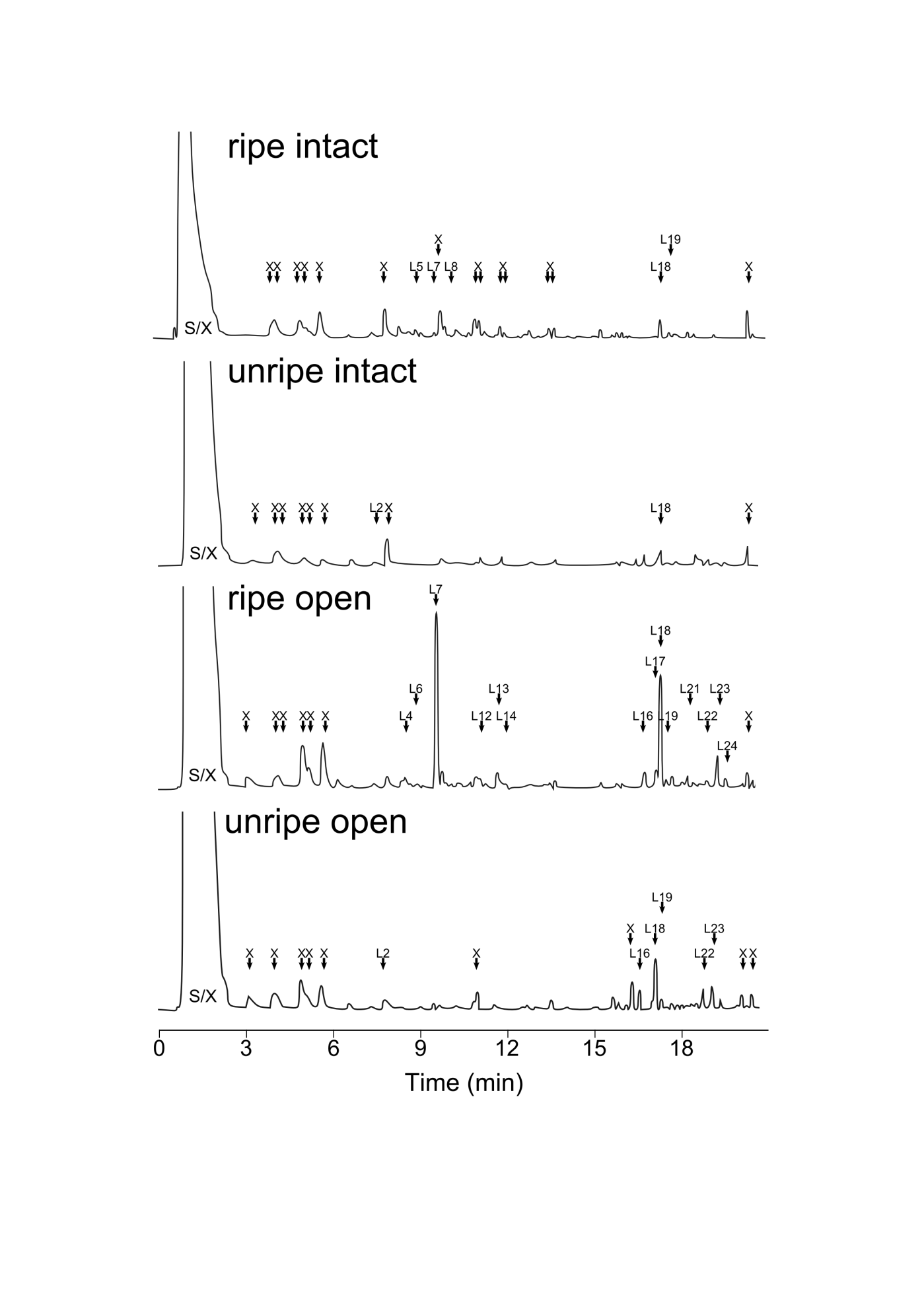


**Fig. S2 – Representative gas chromatograms of odor profiles of *Leonia cymosa*.** Compound names are shown in Tab. S2. X marks contaminants. SX marks a solvent & unknown contaminant peaks present in all samples, including control. Presented samples were chosen according to their proximity to the group means (e.g., ripe intact). However, they may markedly deviate from the group mean with regards to some individual compounds and are thus for illustration only.

**Tab. S3: Compounds identified from *Psychotria cincta.*** Mean (±SD) estimated absolute amounts (mg) of compounds in all *Psychotria cincta* samples in mg after 2.5 h sampling and retention indices (RI). Absolute amounts were calculated based on an external standard. Values are rounded and thus very small amounts are sometimes presented as 0. Compounds presented are those present in at least 8 samples in a species (see Methods). ID method: methods used for identification of the respective compound. RI: retention index matching published values. MS: strong match with published mass spectra. REF: compound identification verified by running a synthetic compound or, in cases of enantiomers and close derivatives, a mixture of known compounds. In unknown compounds, ID method provides dominant MS ions. Compound classes: terpenoids (TRP), aromatic compounds (AR), aldehydes (ALD), alcohols (ALC), esters (EST), acids (AC), ketones (KET)

| **Code** | **ID** | **Class** | **RI** | **ID method** | **Ripe intact** | **Unripe intact** | **Ripe open** | **Unripe open** |
| --- | --- | --- | --- | --- | --- | --- | --- | --- |
| P1 | Unknown C3-benzene | AR | 9.53 | MS | 0.009 ±(0.028) | 0.007 ±(0.018) | 0.04 ±(0.08) | 0 ±(0) |
| P2 | Unknown C3-benzene | AR | 9.63 | MS | 0.003 ±(0.006) | 0.006 ±(0.009) | 0.008 ±(0.017) | 0 ±(0) |
| P3 | 3-Octanone | KET | 9.86 | MS, RI | 0 ±(0) | 0 ±(0) | 0.017 ±(0.034) | 0.021 ±(0.025) |
| P4 | Myrcene | TRP | 9.90 | MS, RI, REF | 0.005 ±(0.016) | 0.004 ±(0.013) | 0.001 ±(0.004) | 0.018 ±(0.039) |
| P5 | Mesitylene | AR | 9.94 | MS, RI | 0.003 ±(0.006) | 0.008 ±(0.011) | 0.007 ±(0.013) | 0 ±(0) |
| P6 | *p*-Cymene | AR, TRP | 10.25 | MS, RI, REF | 0.024 ±(0.075) | 0.009 ±(0.029) | 0 ±(0) | 0.001 ±(0.003) |
| P7 | (*E*)-β−Ocimene | TRP | 10.47 | MS, RI, REF | 0 ±(0) | 0.006 ±(0.013) | 0 ±(0) | 0.009 ±(0.014) |
| P8 | Linalool | TRP, ALC | 11.01 | MS, RI, REF | 0 ±(0) | 0 ±(0) | 0.036 ±(0.045) | 0.243 ±(0.237) |
| P9 | Nonanal | ALD | 11.06 | MS, RI, REF | 0.007 ±(0.009) | 0.009 ±(0.01) | 0.011 ±(0.014) | 0.003 ±(0.006) |
| P10 | α-Copaene | TRP | 13.79 | MS, RI, REF | 0 ±(0) | 0.002 ±(0.003) | 0.001 ±(0.002) | 0.001 ±(0.002) |
|  | TOTAL |  |  |  | 0.05 ±(0.13) | 0.05 ±(0.07) | 0.12 ±(0.16) | 0.3 ±(0. 3) |


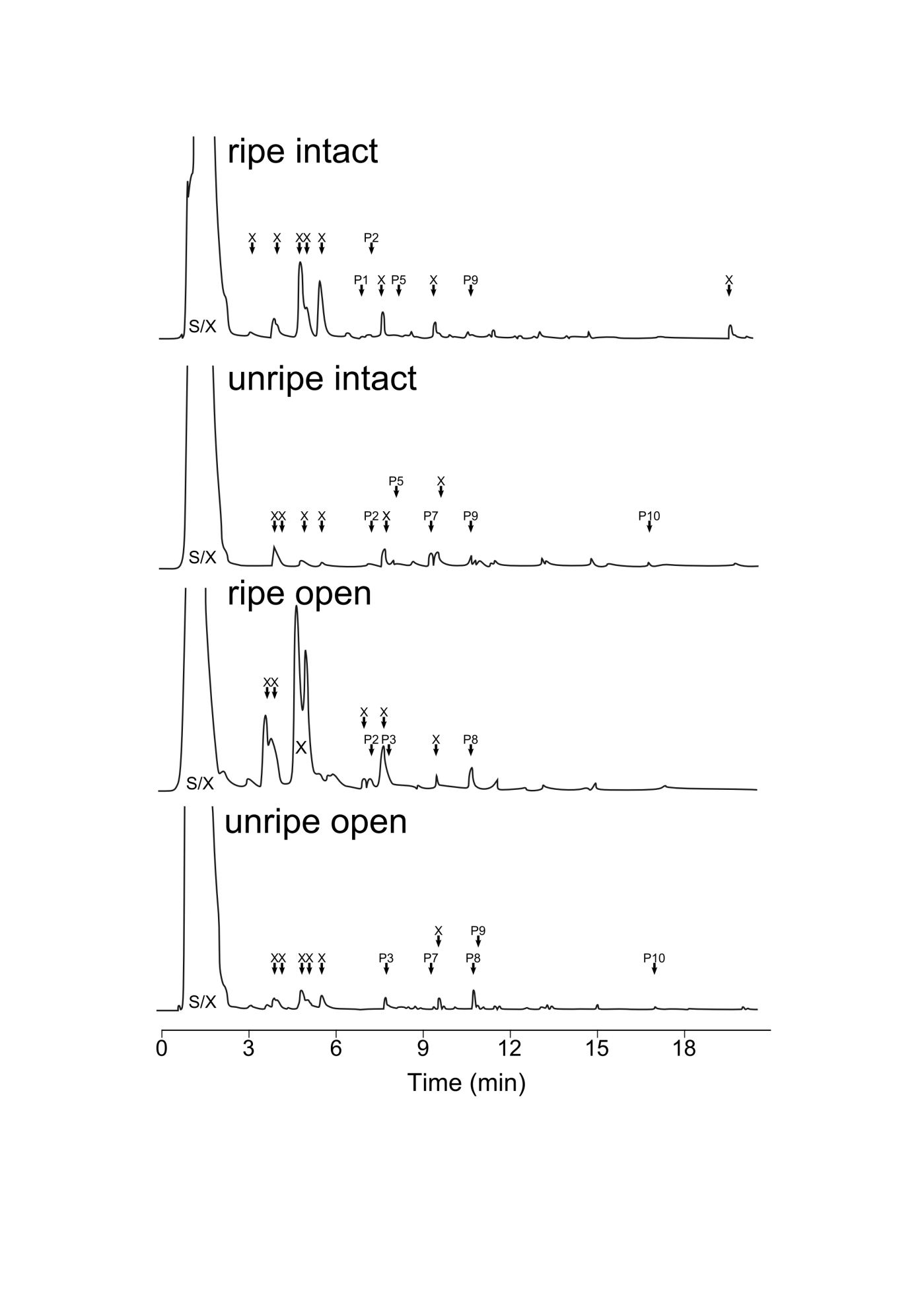


**Fig. S3 – Representative gas chromatograms of odor profiles of *Psychotria cincta*.** Compound names are shown in Tab. S3. X marks contaminants. SX marks a solvent & unknown contaminant peaks present in all samples, including control. Presented samples were chosen according to their proximity to the group means (e.g., ripe intact). However, they may markedly deviate from the group mean with regards to some individual compounds and are thus for illustration only.

**Tab. S4: Compounds identified from *Maieta guianensis.*** Mean (±SD) estimated absolute amounts (mg) of compounds in all *Maieta guianensis* samples in mg after 2.5 h sampling and retention indices (RI). Absolute amounts were calculated based on an external standard. Values are rounded and thus very small amounts are sometimes presented as 0. Compounds presented are those present in at least 8 samples in a species (see Methods). ID method: methods used for identification of the respective compound. RI: retention index matching published values. MS: strong match with published mass spectra. REF: compound identification verified by running a synthetic compound or, in cases of enantiomers and close derivatives, a mixture of known compounds. In unknown compounds, ID method provides dominant MS ions. Compound classes: terpenoids (TRP), aromatic compounds (AR), aldehydes (ALD), alcohols (ALC), esters (EST), acids (AC).

| **Code** | **ID** | **Class** | **RI** | **ID method** | **Ripe intact** | **Unripe intact** | **Ripe open** | **Unripe open** |
| --- | --- | --- | --- | --- | --- | --- | --- | --- |
| M1 | Cumene | AR | 9.23 | MS, RI, REF | 0.025 ±(0.047) | 0.017 ±(0.038) | 0.012 ±(0.039) | 0.023 ±(0.051) |
| M2 | Unknown C3-benzene | AR | 9.53 | MS | 0.017 ±(0.031) | 0.009 ±(0.02) | 0.01 ±(0.033) | 0.015 ±(0.029) |
| M3 | Unknown C3-benzene | AR | 9.62 | MS | 0.006 ±(0.012) | 0.003 ±(0.005) | 0.003 ±(0.011) | 0.007 ±(0.014) |
| M4 | Benzaldehyde | AR, ALD | 9.63 | MS, RI, REF | 0.034 ±(0.096) | 0.031 ±(0.091) | 0.026 ±(0.071) | 0.045 ±(0.108) |
| M5 | 1-Octen-3-one | KET | 9.77 | MS, RI | 0 ±(0) | 0 ±(0) | 0.023 ±(0.025) | 0.014 ±(0.028) |
| M6 | 1-Octen-3-ol | ALC | 9.82 | MS, RI | 0 ±(0) | 0 ±(0) | 0.965 ±(0.822) | 0.356 ±(0.598) |
| M7 | 3-Octanone | KET | 9.86 | MS, RI | 0 ±(0) | 0 ±(0) | 0.216 ±(0.171) | 0.111 ±(0.18) |
| M8 | Mesitylene | AR | 9.94 | MS, RI | 0.008 ±(0.013) | 0.008 ±(0.011) | 0.003 ±(0.011) | 0.007 ±(0.014) |
| M9 | 3-Octanol | ALC | 9.99 | MS, RI | 0 ±(0) | 0 ±(0) | 0.193 ±(0.244) | 0.026 ±(0.049) |
| M10 | *p*-Cymene | AR | 10.25 | MS, RI, REF | 0.006 ±(0.019) | 0.02 ±(0.06) | 0.022 ±(0.07) | 0.024 ±(0.06) |
| M11 | Nonanal | ALD | 11.06 | MS, RI, REF | 0.016 ±(0.011) | 0.002 ±(0.007) | 0.01 ±(0.009) | 0.017 ±(0.011) |
|  | TOTAL |  |  |  | 0.11 ±(0.2) | 0.09 ±(0.21) | 1.49 ±(1.04) | 0.64 ±(0.95) |


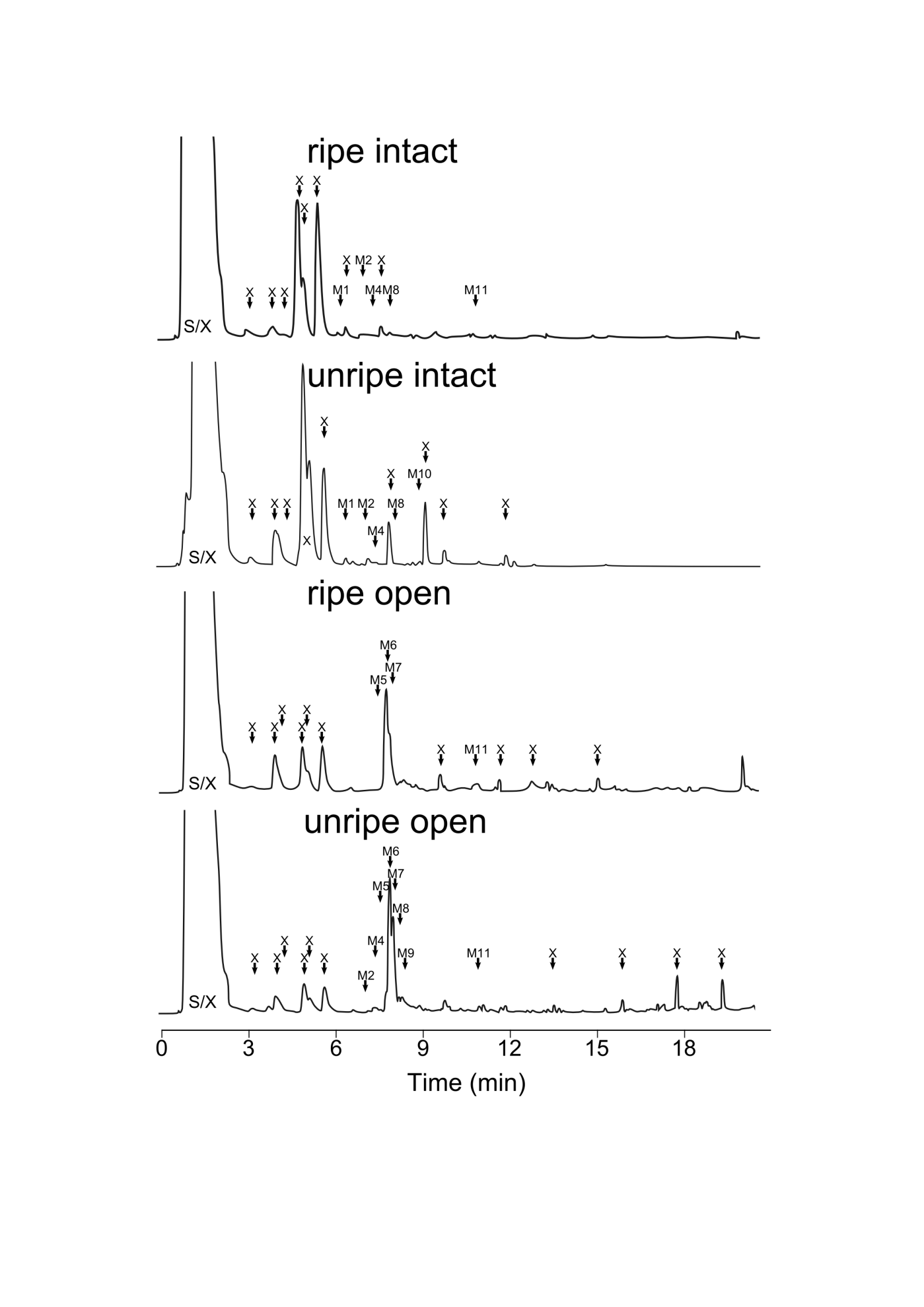


**Fig. S4 – Representative gas chromatograms of odor profiles of *Maieta guianensis*.** Compound names are shown in Tab. S4. X marks contaminants. SX marks a solvent & unknown contaminant peaks present in all samples, including control. Presented samples were chosen according to their proximity to the group means (e.g., ripe intact). However, they may markedly deviate from the group mean with regards to some individual compounds and are thus for illustration only.

**Bibliography – Supp Mat**

Adams RP (2007) Identification of essential oil components by gas chromatography/mass spectrometry, 4th edn. Allured Pub. Corp., Carol Streams, IL

Ballard HE, Paula-souza J De, Wahlert GA (2014) Violaceae. In: Kubitzki K (ed) Flowering plants: Eudicots. Springer, Berlin Heidelberg, pp 303–322

Ballard HE, Sytsma KJ (2000) Evolution and biogeography of the woody Hawaiian violets (Viola, Violaceae): Arctic origins, herbaceous ancestry and bird dispersal. Evolution 54:1521–1532

Bolmgren K, Eriksson O (2005) Fleshy fruits - origins, niche shifts, and diversification. Oikos 109:255–272

Bremer B, Eriksson O (1992) Evolution of fruit characters and dispersal modes in the tropical family Rubiaceae. Biol J Linn Soc 47:79–95.

Clausing G, Meyer K, Renner SS (2000) Correlations among fruit traits and evolution of different fruits within Melastomataceae. Bot J Linn Soc 133:303–326.

Culot L (2009) Primary seed dispersal by two sympatric species of tamarins, Saguinus fuscicollis and Saguinus mystax, and post-dispersal seed fate. Université de Liège, DPZ

Culot L, Huynen MC, Gérard P, Heymann EW (2009) Short-term post-dispersal fate of seeds defecated by two small primate species (Saguinus mystax and Saguinus fuscicollis) in the Amazonian forest of Peru. J Trop Ecol 25:229–238.

Culot L, Muñoz Lazo FJJ, Huynen M-C, Poncin P, Heymann EW (2010) Seasonal variation in seed dispersal by tamarins alters seed rain in a secondary rain forest. Int J Primatol 31:553–569.

Davies TJ, Barraclough TG, Chase MW, Soltis PS, Soltis DE, Savolainen V (2004) Darwin’s abominable mystery: Insights from a supertree of the angiosperms. Proc Natl Acad Sci USA 101:1904–1909

Dötterl S, Jürgens A (2005) Spatial fragrance patterns in flowers of Silene latifolia: Lilac compounds as olfactory nectar guides? Plant Syst Evol 255:99–109.

Dötterl S, Wolfe LM, Jürgens A (2005) Qualitative and quantitative analyses of flower scent in Silene latifolia. Phytochemistry 66:203–213.

Dray S, Dufour AB (2007) The ade4 package: Implementing the duality diagram for ecologists. J Stat Softw 22:1–20

Gorchov DL, Cornejo F, Ascorra C, Jaramillo M (1995) Dietary overlap between frugivorous birds and bats in the Peruvian Amazon. Oikos 74:235–250

Heymann EW (1995) Sleeping habits of tamarins, Saguinus mystax and Saguinus fuscicollos (Mammalia; Primates; Callitrichidae), in north-eastern Peru. J Zool 237:211–226.

Julliot C, Sabatier D (1993) Diet of the red howler monkey (Alouatta seniculus) in French Guiana. Int J Primatol 14:527–550.

Knogge C, Heymann EW (2003) Seed dispersal by sympatric tamarins, Saguinus mystax and Saguinus fuscicollis: Diversity and characteristics of plant species. Folia Primatol 74:33–47.

Peres CA (1994) Diet and feeding ecology of gray woolly monkeys (Lagothrix lagotricha cana) in Central Amazonia: Comparisons with other atelines. Int J Primatol 15:333–372.

Pfrommer A (2009) Seed dispersal ecology of Leonia cymosa (Violaceae) in the rain forest of Eastern Ecuador. Bayrische Julius-Maximilians Universität Würzburg

Quinn GP, Keough MJ (2002) Experimental design and data analysis for biologists. Cambridge University Press, Cambridge

R Core Team (2014) R: A language and environment for statistical computing. R Foundation for Statistical Computing, Vienna, Austria. URL http://www.R-project.org/.

Reinehr A (2010) Seed-dispersal of *Leonia cymosa* (Violaceae) by the sympatric tamarins Saguinus mystax and Saguinus fuscicollis, Primates, Callitrichidae. MSc Thesis. Universität Hohemheim

Renner SS (1989) A survey of reproductive biology in Neotropical Melastomataceae and Memecylaceae. Ann Missouri Bot Gard 76:496–518.

Smith AC (1997) Comparative ecology of saddleback (Saguinus fuscicollis) and moustached (Saguinus mystax) tamarins. University of Reading

Snow DW (1981) Tropical frugivorous birds and their food plants: A world survey. Biotropica 13:1–14.

Stiles FG, Rosselli L (1993) Consumption of fruits of the Melastomataceae by birds: How diffuse is coevolution? In: Fleming TH, Estrada A (eds) Frugivory and Seed Dispersal: Ecological and Evolutionary Aspects. Kluwer Academic Publishers, Dodrecht, pp 57–73

Venables WN, Ripley BD (2002) Modern applied statistics with S. Fourth Edition. Springer, New-York

Wheelwright NT, Haber WA, Murray KG, Guindon C (1984) Tropical fruit-eating birds and their food plants: A survey of a Costa Rican lower montane forest. Biotropica 16:173–192
